# Extravascular Dispersion of Polyvinyl Alcohol Microsphere Particles in Uterine Artery Embolization

**DOI:** 10.1155/2022/7426210

**Published:** 2022-02-15

**Authors:** L. A. Torres-de la Roche, C. Cezar, S. Hanif, R. Devassy, H. Krentel, J. Hennefründ, R. L. De Wilde

**Affiliations:** ^1^University Hospital for Gynecology, Pius-Hospital Oldenburg, University Medicine Oldenburg, Germany; ^2^Department of Laparoscopic Gynecological Minimal-Access Surgery, Dubai London Clinic and Specialty Hospital, UAE; ^3^Clinic of Gynecology, Obstetrics, Oncology and Senology, Bethesda Hospital, Duisburg 47053, Germany

## Abstract

Uterine artery embolization (UAE) is a common minimally invasive treatment of different uterine pathologies, such as fibroids, adenomyosis, and menorrhagia. The procedure involves the injection of embolic agents into the uterine arteries, whereby various particles can be used, such as polyvinyl alcohol (PVA). Complication of UAE is the dispersion of polyvinyl alcohol (PVA) microsphere particles in the uterine body which can lead to a granular vaginal discharge. We report the management of complications of PVA microspheres dispersed from the uterine body causing postprocedural discomfort due to the vaginal passage of microspheres or because of an induced fibroid-size enlargement. The dispersion of the PVA microspheres is one example of a minor UAE complication, which nevertheless causes significant distress to the patient and eventfully requires further surgical interventions.

## 1. Introduction

The injection of microspheres of polyvinyl alcohol (PVA) particles can be successfully used as the minimally invasive procedure to obliterate the vasculature of the uterine body (uterine artery embolization; UAE) in the treatment of different uterine pathologies [1]. UAE is used in young women who desire to preserve their uterus and is minimally invasive and has a faster recovery than most surgical treatments [2]. It may be used as an alternative therapy to myomectomy and hysterectomy, but complications are possible [3, 4]. These events can be classified as major or minor [5] and may result in prolonged hospitalization and troublesome outcomes such as pelvic infection, ischemia, ovarian failure, sexual dysfunction, expulsion of degenerated fibroid tissue, nongynecologic embolization, or pulmonary embolism [5]. Despite the fast recovery time after embolization, the procedure is associated with minor complications and a risk of failure, eventually leading to future necessary surgical intervention [6, 7].

The Society of Obstetricians and Gynaecologists of Canada (SOGC) recommends that patients who consider UAE as a treatment option for symptomatic fibroids be counselled about the early results and the lack of data on the long-term benefits regarding future fertility and pregnancy outcomes [8]. Women should also be counselled regarding the risk of major complications and subsequent hysterectomy. Concerning the satisfaction rates of UAE compared to surgical intervention, no differences were reported after two and five years [4, 8].

Considering that dispersion of polyvinyl alcohol microsphere during UAE is an uncommon event, this paper focuses on the management of this complication presented in patients who underwent UAE because of uterine fibroids, adenomyosis, and menorrhagia.

## 2. Materials and Methods

We retrospectively analyzed the data of patients who presented with complications relating to dispersed PVA microspheres after UAE was performed for different uterine pathologies. The electronic medical records of the patients were evaluated in terms of their clinical symptoms and diagnostic and surgical treatments. The findings are reported according to the PROCESS statement for reporting cases series [9]. Written informed consent to publish from all patients was obtained, and the approval to conduct the present analysis was obtained from the Ethical Committee of the Dubai London Clinic and Speciality Hospital (DLCEC5012021-1; 15.02.2021).

## 3. Results

During the last five year of experience in our clinic, we have found four cases of polyvinyl alcohol microsphere particle dispersion during UAE. In the following, we describe the clinical features of these patients and the management provided.

A 51-year-old patient attended due to symptomatic uterine fibroid and adenomyosis. She complained about menorrhagia and metrorrhagia for five years and received UAE in 2016 that was complicated by extrusion of PVA microspheres. During hysterectomy, it was observed that the parametria were infiltrated, resulting in difficult dissection of the pelvic wall and ureter. Microscopic examination revealed the presence of proteinaceous foreign body (PVA) in the uterine substance and the paratubal soft tissue with granulomatous reaction. The postoperative recovery of the patient was uneventful.

The second case was a 35-year-old patient who was referred for infertility and multiple fibroids, having complaints such as menorrhagia and dysmenorrhea. She was initially given a trial of UAE and concomitant medical management with Ulipristal acetate but failed to respond to the treatment. She had repetitive IVF failures and was referred for myomectomy. Thus, laparoscopic myomectomy was performed which revealed extensive dispersion of PVA particles in the myometrium and in the subcapsular surfaces, requiring thorough rinsing and reconstruction of myometrium. Postoperative recovery was uneventful and patient presented to date symptom free. She was advised to report for follow up in two years.

The third case was a 34-year-old patient that visited the clinic for infertility and multiple myomas, failed UAE, and persistent symptoms of hypermenorrhea. She underwent laparoscopic myomectomy and, thereafter, suffered from repeated in vitro fertilization and embryo transfer (IVF-ET) failures and greenish vaginal discharge. Hysteroscopic resection and evacuation of residual myoma and PVA granules was performed followed by adhesion barrier prophylaxis to prevent synechiae. A second-look hysteroscopy was performed to confirm the absence of foreign bodies in the uterus. The patient had a good postoperative recovery and was planned for embryo transfer (ET).

The fourth case was a 53-year-old patient that visited our hospital for heavy menstrual bleeding due to multiple fibroids. She received both UAE and medical management with ulipristal acetate for symptomatic fibroids which failed to improve the symptoms. Hysteroscopic endometrial resection was performed with the primary intention of biopsy, which revealed PVA particles in the endometrium and subendometrial region encapsulating the fibroids closer to the endometrium. She was advised for follow up after 3 months to evaluate her response to therapy.

### 3.1. Management of Extravascular Dispersion of Polyvinyl Alcohol Microsphere Particles

**Figure 1:**
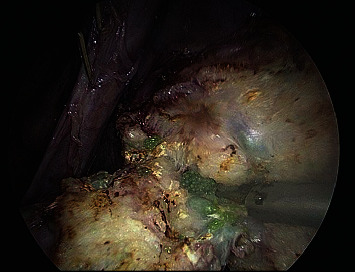
Extrusion of PVA microspheres as observed at laparoscopic supracervical hysterectomy.

**Figure 2:**
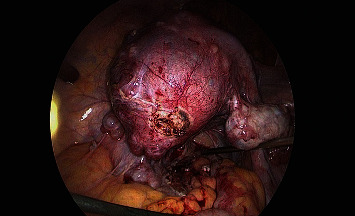
Laparoscopic supracervical hysterectomy in case 1, multiple small myomas visible during operation.

All the aforementioned patients were treated with different treatment modalities following the typical case scenarios in the best interest of the patients. Laparoscopic supracervical hysterectomy was done for the first case with careful preparation of the infiltrated parametrium, and difficult dissection of pelvic wall and ureter, due to extrusion of PVA microspheres (Figure 1**)**. The specimen (Figure 2) was extracted with in-bag morcellation to prevent the spillage of the microspheres into the abdominopelvic cavity. Histopathological analysis revealed benign findings and confirmed PVA particles. No intraoperative or postoperative complications were recorded. Patient was taken into follow up and remained symptom free.

**Figure 3:**
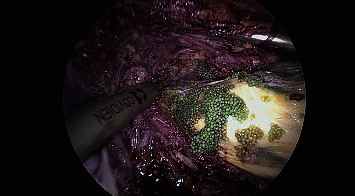
Myomectomy, intraoperative visualization of PVA microspheres.

**Figure 4:**
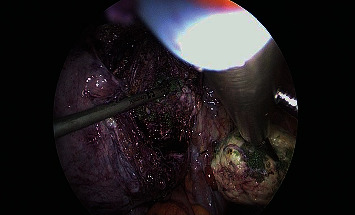
Successful myomectomy.

**Figure 5:**
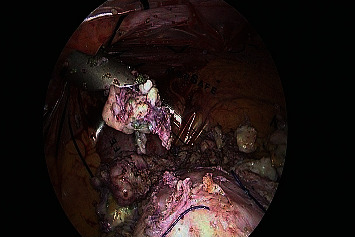
Morcellation of myomas.

The second case was managed by laparoscopic myomectomy. Cytology was obtained of the peritoneal washings followed by careful enucleation of myomatous tissues and the PVA particles in the uterine myometrium (Figure 3). The myometrium was reconstructed with V LOC^R^ suture. The large multiple myomatous specimen was extracted with in-bag morcellation to prevent the spillage of the microspheres into the abdominopelvic cavity (Figures 4 and 5). Histopathological analysis revealed benign findings and confirmed PVA particles. No intra or postoperative complications were recorded. She had symptom-free follow-up.

**Figure 6:**
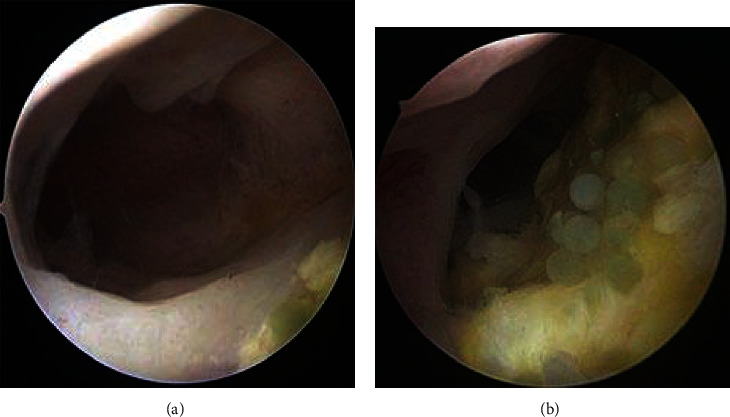
Hysteroscopic aspect of residual myomas and PVA granules. (a) Residual myomas. (b) Polyvinyl alcohol granules.

The third case was treated with hysteroscopic resection of residual myoma, and PVA granules were evacuated (Figure 6). A second-look hysteroscopy confirmed no foreign body in the cavity. Patient was symptom free and was planned for embryo transfer (ET). She was advised for laparoscopic exploration if needed thereafter for residual myomas.

**Figure 7:**
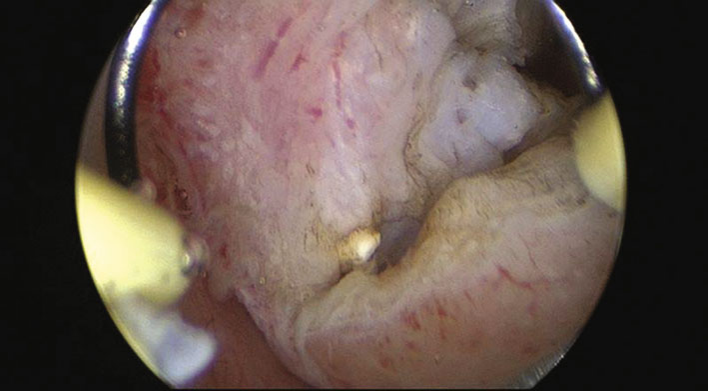
Hysteroscopic resection of the sub-mucous fibroids.

**Figure 8:**
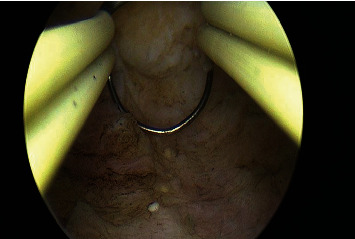
Hysteroscopic resection of endometrium.

The fourth case was treated with hysteroscopic resection of the submucous fibroids (Figure 7). Hysteroscopic endometrial resection was performed with primary intention of biopsy (Figure 8), which revealed the PVA particles in the endometrium and subendometrial region, encapsulating the fibroids closer to the endometrium. She was advised for follow-up after 3 months to evaluate response to therapy.

## 4. Discussion

The use of polyvinyl alcohol (PVA) particles was first reported in 1995 by Ravina et al. in a study of 16 patients treated with UAE for uterine fibroids [10]. On follow-up, 80% of these patients reported resolution of their symptoms, but the others required additional surgery. UAE is associated with shorter hospital stay and better recovery of the patients [5] in comparison to myomectomy or hysterectomy [4]; therefore, UAE can be considered as an alternative to surgery. In our previous publication [5], we found that the patients who benefit most from this therapy are those who are young, suffer from heavy menstrual bleeding resistant to other conservative measures, or have nonpedunculated fibroids, irrespectively of the number and size of fibroids. Pedunculated fibroids are a relative contraindication because of the risk of degeneration and subsequent infection. Regarding the myoma size, aberrant vascularization of large fibroids (>10 cm) should be evaluated before embolization to avoid damage to neighboring abdominal structures. According to a Cochrane Review, involving women wishing to preserve fertility, there is no significant difference between UAE and surgery in patient satisfaction rates at two and five years [4]. Quality-of-life scores were documented better after UAE than high-intensity focused ultrasound ablation (HIFU) [11].

PVA particles have a tendency to clump together to form larger aggregates which can be minimized by their dilution and slow infusion to achieve more distal embolization and sometimes reach the ovarian vessels, potentially affecting the ovarian reserve [12, 13]. Therefore, desired level of occlusion should be determined to select the appropriate particle size to be used. Usually, particles measuring 350–500 or 500–710 *μ* in diameter are used to achieve complete occlusion of uterine arteries that, in turn, induces ischaemic necrosis of the uterine fibroids. However, there is no conclusive evidence about the impact of the blood flow reduction and ionising radiation received during the procedure on fertility and pregnancy. Although loss of ovarian reserve can occur after hysterectomy, myomectomy, and UAE, it occurs more frequently in women older than 45 years that underwent UAE [14]. Other studies report lower pregnancy rates after UAE than after myomectomy [5] or HIFU [11], as well as, higher miscarriage events than after myomectomy [5]. Nonetheless, many confounding factors affect these results, especially younger patients are underrepresented in most of the studies. With reference to complications, no significant difference between UAE and surgery or HIFU has been observed [4, 11].

In one rare complication inherent to UAE, the possibility of PVA dispersion in parametrial and myometrium arteries, as described, can lead to damage to nearby organs, persistent symptoms and even to complete uterine ischemia or endometrial infection requiring hysterectomy [15]. In cases of intracavitary residual myoma, a hysteroscopic PVA granule evacuation and further fibroid resection is a feasible solution to this complication when the uterus is not severely compromised and for patients seeking to get pregnant. For more complicated cases, where the parametrium is distorted or the pelvic wall and ureter are not easily to dissect, laparoscopic supracervical hysterectomy with in-bag morcellation, to prevent the spillage of the microspheres into the abdominopelvic cavity, could be performed. In accordance to our experience, the acute management of this rare complication should be individualized, taking in consideration the clinical situation and patient's desire.

In addition to proper technique during the UAE procedure, adequate patient selection is crucial to avoid the aforementioned complication and further interventions [5, 16]. With regard to improving fertility chances after UAE, there are no actual data to support this issue [5], and women should be informed before the procedure [17, 18]. For symptomatic fibroids, UAE may be successfully used as an alternative to hysterectomy or myomectomy, especially in women with desire to preserve their uterus [5]; however, the risk of complications must be discussed with the patients before initiating the therapeutic plan [5].

## 5. Conclusions

Although UAE is a commonly practiced procedure for the treatment of uterine fibroids specially to preserve the uterus, minor complications have been reported. The dispersion of the PVA microspheres is one of those minor complications which are apparently rare but can cause significant distress to the patient and require further surgical interventions.

The proper selection of cases to receive UAE should be carried out, and a therapeutic plan of the uterus pathology should be established together with the patients, taking into account the current evidence-based data for UAE, therapeutic goals to be achieved, and, last but not least, the possibility of minor and major complications of the procedure. The acute management of complications should be individualized in accordance to the clinical situation and patient's desire.

## Data Availability

The clinical data used to support the findings of this study are stored at Department of Laparoscopic Gynecological Minimal-Access Surgery, Dubai London Clinic and Specialty Hospital, United Arab Emirates and are available from corresponding author upon request.

## References

[1] Duvnjak S., Ravn P., Green A., Andersen P. (2017). Uterine fibroid embolization with acrylamido polyvinyl microspheres: prospective 12-month clinical and MRI follow-up study. *Acta Radiologica*.

[2] Masciocchi C., Arrigoni F., Ferrari F. (2017). Uterine fibroid therapy using interventional radiology mini-invasive treatments: current perspective. *Medical Oncology*.

[3] Toor S., Jaberi A., Macdonal D., MDF M., Schweitzer M., Rasuli P. (2012). Complication rates and effectiveness of uterine artery embolization in the treatment of symptomatic Leiomyomas: a systematic review and meta-analysis. *AJR*.

[4] Gupta J., Sinha A., Lumsden M., Hickey M., Cochrane Gynaecology and Fertility Group (2014). Uterine artery embolization for symptomatic uterine fibroids. *Cochrane Database of Systematic Reviews*.

[5] Cezar C., Torres de la Toche L., Hennefründ J., Verhoeven H., Devassy R., DeWilde R. (2021). Can uterine artery embolization be an alternative to plastic and reconstructive uterus. *GMS interdisciplinary plastic and reconstructive surgery DGPW*.

[6] Martin J., Bhanot K., Athreya S. (2013). Complications and reinterventions in uterine artery embolization for symptomatic uterine fibroids: a literature review and meta analysis. *Cardiovascular and Interventional Radiology*.

[7] Kohi M., Spies J. (2018). Updates on uterine artery embolization. *Seminars in Interventional Radiology*.

[8] SOGC clinical practice guidelines (2005). Uterine fibroid embolization (UFE). *International Journal of Gynaecology and Obstetrics: The Official Organ of the International Federation of Gynaecology and Obstetrics*.

[9] Agha R., Sohrabi C., Mathew G. (2020). The PROCESS 2020 guideline: updating consensus preferred reporting of case series in surgery (PROCESS) Guidelines. *International Journal of Surgery*.

[10] Ravina J., Ciraru-Vigneron N., Bouret J. (1995). Arterial embolisation to treat uterine myomata. *The Lancet*.

[11] Liu L., Wang T., Lei B. (2021). Uterine artery embolization compared with high-intensity focused ultrasound ablation for the treatment of symptomatic uterine myomas: a systematic review and meta-analysis. *Journal of Minimally Invasive Gynecology*.

[12] Goodwin S., Vedantham S., Mc Lucas B., Forno A., Perrella R. (1997). Preliminary experience with uterine artery embolization for uterine fibroids. *Journal of Vascular and Interventional Radiology*.

[13] Shlansky-Goldberg R., Rosen M., Mondschein J., Stavropoulos S., Trerotola S., Diaz-Cartelle J. (2014). Comparison of polyvinyl alcohol microspheres and tris-acryl gelatin microspheres for uterine fibroid embolization: results of a single-center randomized study. *Journal of Vascular and Interventional Radiology*.

[14] Pérez-López F. R., Ornat L., Ceausu I. (2014). EMAS. EMAS position statement: management of uterine fibroids. *Maturitas*.

[15] Aziz A., Petrucco O., Makinoda S. (1998). Transarterial embolization of the uterine arteries: patient reactions and effects on uterine vasculature. *Acta Obstetricia et Gynecologica Scandinavica*.

[16] Joyce A., Hessami S., Heller D. (2001). Leiomyosarcoma after uterine artery embolization. A case report. *The Journal of Reproductive Medicine*.

[17] McLucas B., Voorhees W., Elliott S. (2016). Fertility after uterine artery embolization: a review. *Minimally Invasive Therapy*.

[18] Karlsen K., Hrobjartsson A., Korsholm M., Mogensen O., Humaidan P., Ravn P. (2018). Fertility after uterine artery embolization of fibroids: a systematic review. *Archives of Gynecology and Obstetrics*.

